# Ovarian stimulation with human and recombinant gonadotropin – comparison of in vitro fertilization efficiency with use of time-lapse monitoring

**DOI:** 10.1186/s12978-015-0106-8

**Published:** 2015-12-15

**Authors:** Artur Wdowiak, Iwona Bojar

**Affiliations:** Diagnostic Techniques Unit, Faculty of Health Sciences, Medical University, ul. Staszica 4/6, 20-081 Lublin, Poland; Department for Health Problems of Ageing, Institute of Rural Health, Lublin, Poland; International Scientific Association for the Support and Development of Medical Technologies, Lublin, Poland

**Keywords:** In vitro fertilization (IVF), Intracytoplasmic sperm injection (ICSI), Ovarian stimulation, Embryo development dynamics, Follicle-stimulating hormone, Anti-Müllerian hormone, Estradiol, Time-lapse monitoring

## Abstract

**Background:**

Achieving pregnancy by in vitro fertilization (IVF) treatment depends on many factors, including the ovaries’ capacity and the efficiency of ovarian stimulation. The aim of this study was to assess the influence of ovarian stimulation with human and recombinant gonadotropin, as well as specific hormonal parameters, on the effectiveness of IVF and the dynamics of embryonic development.

**Methods:**

The study involved 221 women aged 25–35 years in whom intracytoplasmic sperm injection was performed. The ovarian stimulation was carried out according to the short protocol: injections of gonadotropin-releasing hormone analogue were followed by human (hFSH) and recombinant (rFSH) follicle-stimulating hormone administration. The growth of embryos was monitored with a time-lapse system. Levels of follicle-stimulating hormone (FSH), luteinizing hormone (LH), and anti-Müllerian hormone (AMH) were measured before ovarian stimulation, and levels of estradiol were assessed on the day of administration of recombinant chorionic gonadotropin.

**Results:**

Pregnancy was achieved in 77 women (group A) – 42 (54.55 %) of them were stimulated with hFSH and 35 (45.45 %) were stimulated with rFSH. Among the 144 women in whom pregnancy was not achieved (group B), hFSH was administered to 73 (50.69 %) women and rFSH to 71 (49.31 %) women. In both groups subsequent embryo development stages were usually noted earlier after hFSH stimulation than after rFSH stimulation. The average values of AMH, estradiol, and estradiol per >17 mm follicle were higher in group A; in turn, FSH and LH mean levels were higher in group B. ROC curve analysis showed no statistically significant differences between accuracy of using FSH and AMH levels to predict pregnancy after IVF.

**Conclusions:**

The kind of gonadotropin applied to stimulate ovaries impacts the dynamics of embryo development - in women stimulated with hFSH, subsequent development stages were usually observed earlier than in women treated with rFSH; however, there was no statistically significant difference in pregnancy rates between women who were hFSH stimulated and those who were rFSH stimulated. The mean estradiol level was higher in women who achieved pregnancy than in women in whom pregnancy was not achieved AMH and FSH have the greater impact on achieving pregnancy than other hormones, and the value of AMH and FSH in predicting pregnancy is similar.

## Background

Infertility is recognized as a social disease, and its prevalence is estimated to be around 9 % worldwide for women in reproductive age. The method the most often used to treat infertility is in vitro fertilization (IVF) with intracytoplasmic sperm injection (ICSI). This method gives the highest success rate per cycle in comparison with other treatment options [1]. As a preparation for IVF/ICSI, ovarian stimulation is performed in order to induce the development of multiple follicles of the ovaries. Then, oocytes are aspirated, injected with sperm, and placed into special medium. After a few days of culture, the embryos regarded as the best-developed are transferred into the woman’s uterus [2]. Achieving pregnancy by means of IVF/ICSI treatment depends on many factors, including the capacity of the ovaries and the performed ovarian stimulation. The ovarian reserve is assessed i.a. on the basis of gonadotropins (follicle-stimulating hormone – FSH and luteinizing hormone - LH) and anti-Müllerian hormone (AMH) levels [3]. Ovarian stimulation may be performed with use human or recombinant FSH; however, it is still not clear which kind of gonadotropin gives better IVF outcomes [4–7]. On the other hand, although estrogens and progesterone have been identified as the key hormones involved in the course of oocyte maturation, which appears to be strongly linked to a successful result in assisted reproduction treatment, it has been not clarified whether or not they affect the probability of pregnancy [8, 9]. IVF/ICSI outcomes may also be influenced by proper selection of the embryo to transfer. For a long time embryos were evaluated based on morphological features. Recently, there has been a notable increase in monitoring embryos in real time using a camera placed inside the incubator. A time-lapse embryo monitoring system enables observation of all embryos without any disturbance in incubation conditions or alteration of daily routine [10–13]. The embryo growth is observed at fixed times, and those that show better dynamics of development are regarded as the most suitable for transfer [14].

The aim of this study was to assess the influence of ovarian stimulation with human and recombinant gonadotropin, as well as specified hormonal parameters, on the effectiveness of IVF and the dynamics of embryo development.

## Methods

### Studied population

The presented study was conducted in the years 2013–2014 in the “Ovum Reproduction and Andrology” Non-Public Health Care Unit in Lublin (Poland). The study involved 223 women undergoing IVF treatment for the first time. The inclusion criteria were: age below 35 years, FSH level ≤10 mIU/mL, AMH level ≥1.5 ng/mL, and Body Mass Index (BMI) ≤30 kg/m^2^. Women with severe endometriosis, metabolic disease, or leiomyoma were excluded from the study. In all women ICSI was performed due to abnormal semen parameters. In two women embryo development stopped before achieving blastocyst stage, and therefore they were excluded from the study. Finally, analysis was made for the 221 women in whom embryos achieved the blastocyst stage and were transferred into uterus.

### ICSI procedure

**Fig. 1:**
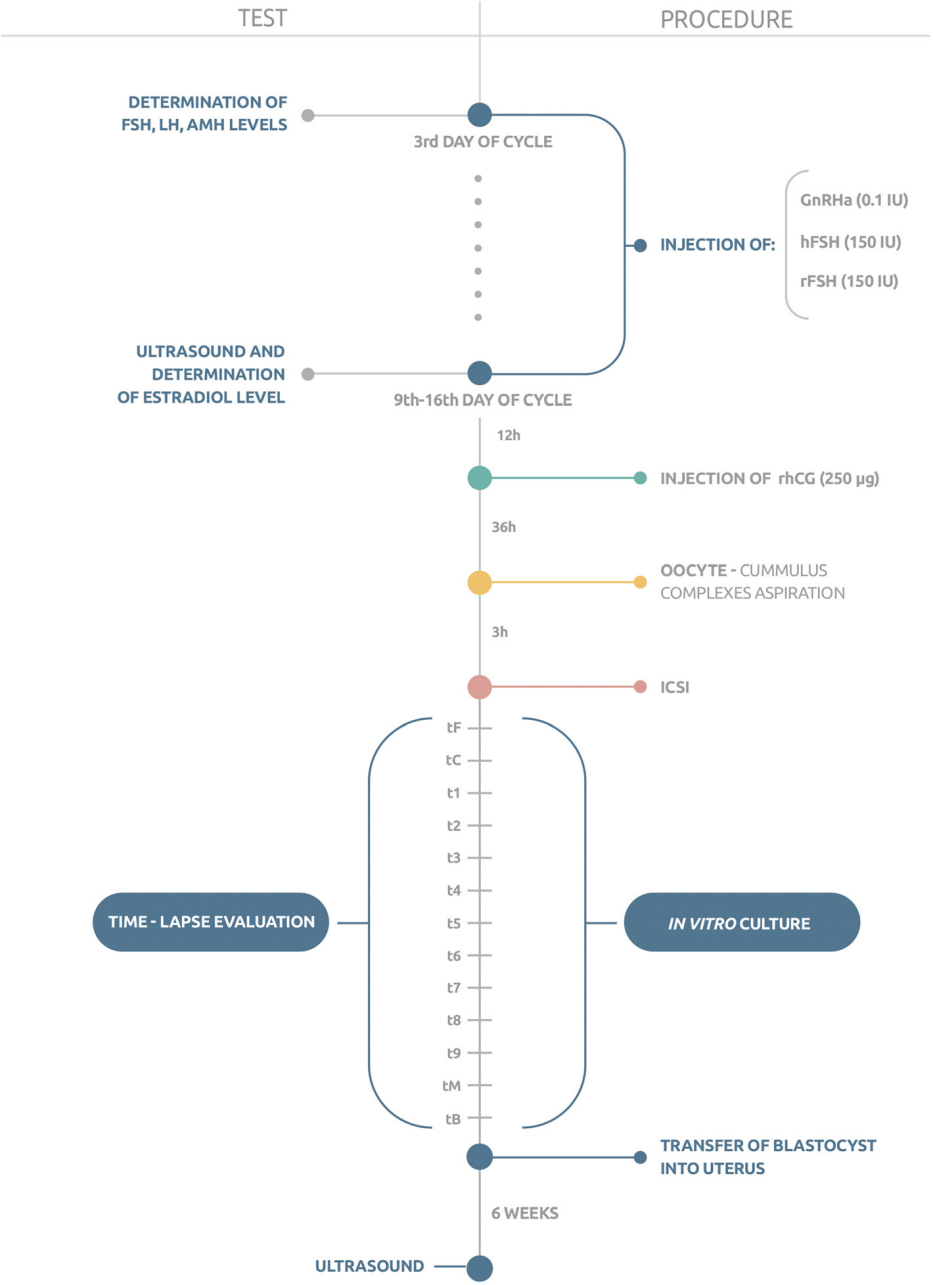
Methodological scheme of the study. (FSH – follicle-stimulating hormone, LH – luteinizing hormone, AMH – anti-Müllerian hormone, hFSH – human follicle-stimulating hormone; rFSH – recombinant follicle-stimulating hormone; GnRHa – gonadotropin-releasing hormone analogue, rhCG – recombinant human chorionic gonadotropin; ICSI – intracytoplasmic sperm injection; tF – time of the first frame in which both pronuclei could be observed; tC – the frame with the last observation of both pronuclei; t1-t9 – times for corresponding numbers of cells; tM – the first frame in which the embryos were compacting into the morula stage; tB – the frame in which a crescent-shaped area began to emerge from the morula)

The ovarian stimulation was carried out according to a standard ovarian stimulation protocol [2]. The short version protocol was used because this procedure is the most common in Poland due to reimbursement by the Polish Government only of short-acting drugs. The methodological part of the study is shown in Fig. 1. In all women injections of gonadotropin-releasing hormone analogue (GnRH) (Diphereline®: Ipsen Pharma) in a dose 0.1 IU were implemented from the first day of the menstrual cycle. From third day of cycle women had also daily injections of FSH in a dose 150 IU. Two types of FSH were randomly administered: human FSH extracted from the urine of post-menopausal women (hFSH; Fostimon®: IBSA) was given to 115 women, and recombinant FSH (rFSH; Gonal-F®: Merck-Serono) was applied to 106 women. hFSH and rFSH was administered until the 9^th^-16^th^ day of cycle when follicles with dimensions equal to or more than 17 mm were found and serum estradiol levels adequate to the number of follicles (over 150 pg/mL per follicle equal to or greater than 17 mm) were noted. Subsequently, all women were injected with 250 μg of recombinant human chorionic gonadotropin (rhCG; Ovitrelle®: Merc-Serono). After 36 h vaginal ultrasound-guided aspiration of oocyte-cumulus complexes was performed and oocytes were placed in a fertilization medium (COOK, Sydney IVF, Australia) under mineral oil. Three hours after retrieval up to six oocytes were subjected to denudation and ICSI procedure. Then, the in vitro culture was carried out until day 2 (2–5 cells stage) in 25 μL of Cleavage medium (COOK, Sydney IVF, Australia) under mineral oil in automated incubators with 5 % CO_2_ at 37 °C. Fifty hours from ICSI the in vitro culture media was changed to Blastocyst medium (COOK, Sydney IVF, Australia).

### Time-lapse evaluation

The growth of all the embryos was monitored continuously by obtaining images at 10-minute intervals. This imaging was achieved with use of a compact time-lapse microscope system (Primo Vision EVO Microscope, Cryo-Innovation, Hungary) placed inside incubator combined with a microwell embryo culture dish. During the time-lapse observation, the embryos were not moved. Between image acquisitions, the system was turned off completely to avoid exposure of the embryos to electromagnetic radiation.

The following terms for the timing of the stages of embryo growth were adopted: t0 - the time when ICSI was carried out; tF - the time of the first frame in which both pronuclei could be observed; tC - the time of the frame with the last observation of both pronuclei; t1 – the time when one cell stage was observed; t2, t3, t4, t5, t6, t7, t8, and t9 - the stages for the corresponding number of cells, e.g. t2 for 2 cells, t3 for 3 cells, etc. (stages were annotated at the first frame in which the cells were seen as separated by membranes); tM - the time of the first frame in which the embryos were compacting into the morula stage; tB - the time of the frame in which a crescent-shaped area began to emerge from the morula; and tEB – the time of the frame with expanded blastocyst with increased volume and expansion of the blastocoele cavity.

Based on the analysis of time-lapse records and in accordance with American Society for Reproductive Medicine and the European Society of Human Reproduction and Embryology consortium guidelines [15], a single blastocyst was selected to be transferred into the uterus. After 6 weeks, the presence of the embryo and its cardiac activity were assessed via ultrasound.

### Determination of hormone levels and imaging studies

Levels of FSH, LH, and AMH were determined on the third day of the cycle preceding ovulation, before administration of drugs. Ultrasound examination was performed daily since 9th day of cycle and after observation of a follicle with dimension equal to or greater than 17 mm; daily measurement of estradiol levels was implemented. The estradiol values were then converted into the levels of estradiol per follicle equal to or greater than 17 mm (E2/f). All hormones levels were measured in serum obtained from morning blood samples (5 mL). Levels of FSH, LH, and estradiol were assessed with electrochemiluminescent method on a Cobas analyzer (Roche Diagnostics) – the reference values were: for FSH 3.5–12.5 mIU/mL; for LH 2.4–12.6 mIU/mL; and for estradiol 12.5–166 pg/mL. Levels of AMH were measured with AMH Gen II ELISA test (Beckman Coulter) on a Euroimmun analyzer (reference range > 1.5 ng/mL).

### Statistical analysis

The obtained results were subjected to the Statistica 9.1 software system (StatSoft, Poland). The measurable parameters were reported as mean (M), standard deviation (SD), and minimum (Min) or maximum (Max) whereas the values of the immeasurable parameters were reported as proportion and quantity. For quality attributes, the Chi^2^ test was performed to show differences between the examined groups. The Shapiro-Wilk normality test was used to check the normality of distribution of variables in the examined groups. The Mann-Whitney U-test was carried out to examine differences between the groups. The r-Pearson correlation test was used to check the correlations between variables. The receiver operating characteristic (ROC) curve was used to assess diagnostic values of the tested parameters. The significance level was set at *p* <0.05, which indicates the existence of statistically significant differences or correlations.

### Ethical statement

The studies were approved by the Ethics Committee of the Institute of Rural Health in Lublin. All women were provided with oral and written information about the study and signed a written consent allowing the use of their data for research purposes.

## Results

### Characteristics of the studied population and the effectiveness of IVF

**Fig. 2:**
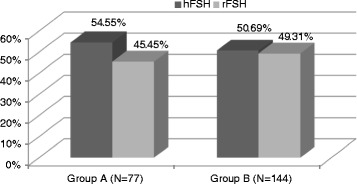
Comparison of gonadotropins administration in the group of women who achieved pregnancy and in the group of women in whom pregnancy was not achieved. (Group A – women who achieved pregnancy; Group B – women in whom pregnancy was not achieved; hFSH – human follicle-stimulating hormone; rFSH – recombinant follicle-stimulating hormone) (Chi^2^ = 0.298, df = 1, p = 0.585)

The mean age of the women included in the study was 30.90 ± 2.93 years and ranged from 25 to 35 years. In turn, the BMI of the studied women was 22.88 ± 3.27 kg/m^2^ and ranged from 17 to 30 kg/m^2^. The women were divided into two groups according to the results of IVF. Group A consisted of 77 women (34.8 %) who achieved pregnancy, and group B was made up of 144 women (65.2 %) in whom pregnancy has not been achieved. In group A, 42 women (54.55 %) were stimulated with hFSH and 35 women (45.45 %) with rFSH. In group B, hFSH was administered to 73 women (50.69 %) and rFSH to 71 women (49.31 %) (Fig. 2). No statistically significant difference was found between the two groups with regards to the type of gonadotropins used (Chi^2^ = 0.298, df = 1, *p* = 0.585). Detailed information about the studied population is presented in Table 1.

**Table 1:** Baseline characteristics of the entire studied population and groups

		Group A	Group B	Whole population
		*n* = 77	*n* = 144	*n* = 221
Age [years]	M ± SD	30.70 ± 3.06	31.0 ± 2.68	30.90 ± 2.93
	Min – Max	25 - 35	25 - 35	25 - 35
BMI [kg/m2]	M ± SD	22.58 ± 3.12	23.04 ± 3.34	22.88 ± 3.27
	Min – Max	17 - 30	17 - 30	17 - 30
Number of women stimulated with hFSH		42	73	115
Number of women stimulated with rFSH		35	71	106

(*Group A* – women who achieved pregnancy, *Group B* women in whom pregnancy was not achieved, *BMI* body mass index, *hFSH* human follicle-stimulating hormone, *rFSH* recombinant follicle-stimulating hormone, *M* mean, *SD* standard deviation, *Min* minimum, *Max* maximum)

### Time-lapse evaluation

**Fig. 3:**
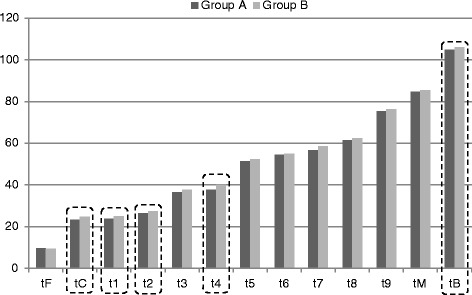
Timing of subsequent embryo development stages in the group of women who achieved pregnancy and in the group of women in whom pregnancy was not achieved. (data are expressed as mean time in hours; time stages statistically significantly different were marked with dotted lines; Group A – women who achieved pregnancy; Group B – women in whom pregnancy was not achieved; tF – time of the first frame in which both pronuclei could be observed; tC – the frame with the last observation of both pronuclei; t1-t9 – times for corresponding numbers of cells; tM – the first frame in which the embryos were compacting into the morula stage; tB – the frame in which a crescent-shaped area began to emerge from the morula)

Comparing time of embryo development in groups A and B it was noted that almost all successive stages of embryo development (except tF) occurred earlier in women who achieved pregnancy than in women in whom pregnancy was not achieved. However, statistically significant differences were found only between the following time stages: tC (Z = −2.705, p = 0.007), t1 (Z = −2.483, p = 0.013), t2 (Z = −1.985, *p* = 0.047), t4 (Z = −3.702, *p* <0.001), and tB (Z = −2.902, *p* = 0.004) (Fig. 3).

**Fig. 4:**
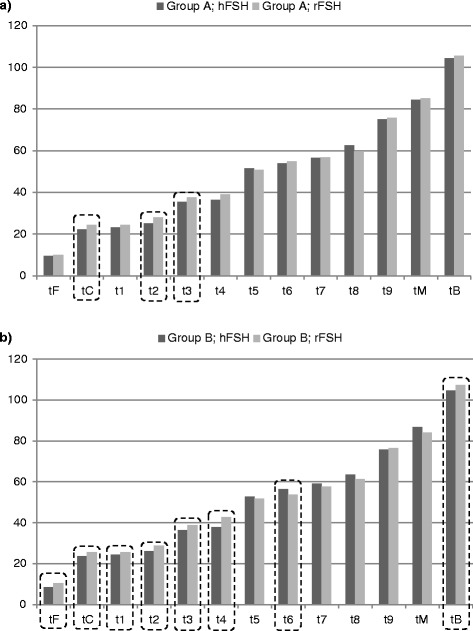
Timing of subsequent embryo development stages depending on the type of gonadotropin used for ovarian stimulation in the group of women who achieved pregnancy (**a**) and in the group of women in whom pregnancy was not achieved (**b**). (data are expressed as mean time in hours; time stages statistically significantly different were marked with dotted lines; Group A – women who achieved pregnancy; Group B – women in whom pregnancy has not been achieved; tF – time of the first frame in which both pronuclei could be observed; tC – the frame with the last observation of both pronuclei; t1-t9 – times for corresponding numbers of cells; tM – the first frame in which the embryos were compacting into the morula stage; tB – the frame in which a crescent-shaped area began to emerge from the morula)

Moreover, in groups A and B the timing of embryo development stages achieved via hFSH and rFSH administration was assessed (Fig. 4). In group A, all stages except t5 and t8 were achieved faster in women stimulated with hFSH than in women stimulated with rFSH, but the differences were statistically significant only for time stages tC (Z = −2.653, *p* = 0.008), t2 (Z = −2.943, *p* = 0.003), and t3 (Z = −2.099, *p* = 0.036).

In group B, faster embryo development after hFSH than after rFSH stimulation was observed for time stages: tF, tC, t1, t2, t3, t4, t9, and tB, and statistically significant differences were found for all these time stages except for t9: tF (Z = −3.572, *p* <0.001), tC (Z = −3.612, *p* <0.001), t1 (Z = −2.322, *p* = 0.020), t2 (Z = −3.823, *p* <0.001), t3 (Z = −3.512, *p* <0.001), t4 (Z = −3.929, *p* <0.001), and tB (Z = −4.133, *p* <0.001). Additionally, a statistically significant difference was noted for t6 stage that was recorded earlier for rFSH-stimulated embryos than for embryos stimulated with hFSH (Z = 2.026, *p* = 0.043).

### Evaluation of hormone levels

**Fig. 5:**
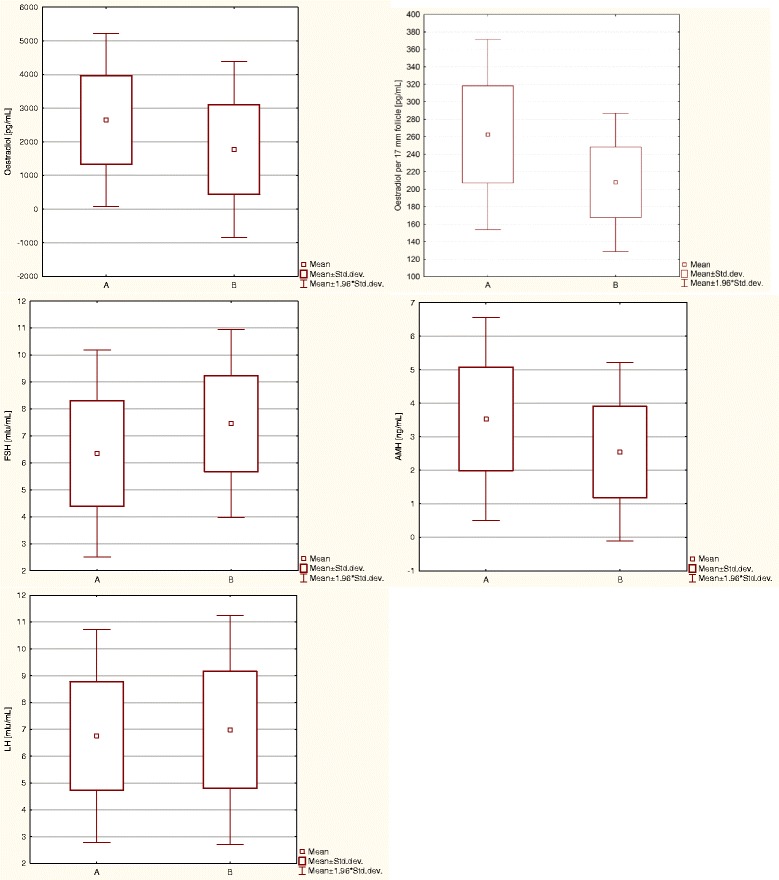
Average hormone levels in the group of women who achieved pregnancy and in thegroup of women in whom pregnancy was not achieved. (Group A – women who achieved pregnancy; Group B – women in whom pregnancy was not achieved; FSH – follicle-stimulating hormone; LH – luteinizing hormone; AMH – anti-Müllerian hormone)

The average values of FSH and LH were higher in group B; in turn, levels of AMH, estradiol, and E2/f were higher in group A (Fig. 5). The level of E2/f was statistically significant different between groups A and B (Z = 6.782, *p* <0.001). Similarly, estradiol (Z = 4.905, *p* <0.001), FSH (Z = −4.686, *p* <0.001), and AMH (Z = 5.024, *p* <0.001) levels differed significantly between the two groups. However, differences in LH levels (Z = −0.623, *p* = 0.533) were not statistically significant (Table 2).

**Table 2:** Comparison of mean hormone levels in the group of women who achieved pregnancy and in the group of women in whom pregnancy was not achieved

	Group A (*n* = 77)		Group B (*n* = 144)		Z	*p*
	M	SD	M	SD		
E2/f [pg/mL]	262.61	55.50	207.88	40.38	6.782	<0.001
FSH [mIU/mL]	6.35	1.96	7.73	1.84	−4.686	<0.001
AMH [ng/mL]	3.26	1.83	2.06	1.62	5.024	<0.001
Estradiol [pg/mL]	2648.66	1310.89	1770.88	1335.51	4.905	<0.001
LH [mIU/mL]	6.75	2.03	6.98	2.18	−0.623	0.533

(Z - Mann Whitney’s test result, *p* <0.05; *Group A* women who achieved pregnancy, *Group B* women in whom pregnancy was not achieved, *E2/f* estradiol per ≥17 mm follicle, *FSH* follicle-stimulating hormone, *LH* luteinizing hormone, *AMH* anti-Müllerian hormone, *M* mean, *SD* standard deviation)

### Correlations between hormone levels and time stages of embryo development

The linear relationship between the various hormone levels and the periods of embryo development was assessed based on Pearson’s correlation coefficient (r) (Table 3). In group A, average-level negative correlations were found between AMH levels and t2 (r = −0.331, *p* = 0.003) and tB (*r* = −0.351, *p* = 0.002), and a low-level negative correlation between AMH levels and stage t4 (*r* = −0.232, *p* = 0.042) was shown. A weak positive correlation was observed between FSH levels and tB stage (*r* = 0.251, *p* = 0.028). Weak correlations were found between LH levels and development stages as follows: negative in t8 (*r* = −0.247, *p* = 0.030) and positive in t9 (r = 0.270, p = 0.018). For E2/f, no statistically significant correlations with embryo development stages were found. However, negative correlations for estradiol levels were observed in the following stages: t2 (*r* = −0.294′, *p* = 0.010) (low level), t4 (*r* = −0.363, *p* = 0.001) (average level), t5 (*r* = −0.292, *p* = 0.010) (slight level), and tB (*r* = −0.284, *p* = 0.012) (slight level).

**Table 3:** Correlations between hormone levels and time stages of embryo development in women who achieved pregnancy and in women in whom pregnancy was not achieved

		tF	tC	t1	t2	t3	t4	t5	t6	t7	t8	t9	tM	tB
Group A (*n* = 77)														
E2/f [pg/mL]	r	−0.157	0.026	−0.098	−0.038	0.096	−0.220	−0.146	−0.048	0.074	−0.043	−0.098	0.220	−0.035
	p	0.172	0.823	0.395	0.744	0.406	0.055	0.206	0.676	0.522	0.710	0.397	0.055	0.760
FSH [mIU/mL]	r	−0.087	−0.150	0.024	0.088	−0.076	0.080	0.041	0.088	−0.066	0.005	0.117	0.043	0.251
	p	0.454	0.194	0.837	0.446	0.512	0.492	0.726	0.446	0.570	0.965	0.310	0.710	0.028
AMH [ng/mL]	r	−0.118	−0.154	−0.085	−0.331	−0.111	−0.232	−0.119	−0.173	−0.093	0.142	−0.013	−0.019	−0.351
	p	0.306	0.180	0.460	0.003	0.336	0.042	0.301	0.133	0.422	0.219	0.912	0.871	0.002
Estradiol [pg/mL]	r	−0.085	−0.131	−0.193	−0.294	−0.169	−0.363	−0.292	−0.130	0.116	0.093	−0.043	0.056	−0.284
	p	0.464	0.258	0.093	0.010	0.141	0.001	0.010	0.259	0.316	0.419	0.709	0.626	0.012
LH [mIU/mL]	r	−0.024	−0.215	−0.102	0.084	−0.059	−0.076	−0.097	0.001	0.011	−0.247	0.270	0.103	0.117
	p	0.839	0.061	0.377	0.470	0.609	0.514	0.400	0.994	0.922	0.030	0.018	0.371	0.312
Group B (*n* = 144)														
E2/f [pg/mL]	r	−0.034	−0.074	−0.121	0.017	−0.082	−0.151	−0.091	0.050	0.111	0.254	0.123	0.024	−0.086
	p	0.685	0.376	0.149	0.844	0.330	0.071	0.281	0.551	0.185	0.002	0.143	0.774	0.305
FSH [mIU/mL]	r	0.078	−0.043	0.041	0.117	0.125	0.150	−0.143	0.150	0.017	−0.155	−0.046	0.131	0.281
	p	0.352	0.611	0.627	0.164	0.134	0.073	0.087	0.074	0.837	0.064	0.584	0.118	0.001
AMH [ng/mL]	r	−0.107	−0.132	−0.197	−0.149	−0.221	−0.237	−0.051	−0.185	0.036	0.085	−0.080	−0.048	−0.279
	p	0.200	0.114	0.018	0.074	0.008	0.004	0.542	0.026	0.673	0.313	0.342	0.566	0.001
Estradiol [pg/mL]	r	−0.214	−0.100	−0.131	−0.072	−0.166	−0.225	−0.075	−0.142	0.074	0.068	−0.005	−0.015	−0.234
	p	0.010	0.232	0.118	0.393	0.047	0.007	0.370	0.091	0.379	0.417	0.953	0.863	0.005
LH [mIU/mL]	r	−0.190	−0.070	0.043	−0.047	0.077	0.062	0.040	0.001	−0.009	0.045	0.133	−0.085	0.068
	p	0.023	0.405	0.613	0.578	0.362	0.457	0.637	0.990	0.916	0.596	0.113	0.313	0.417

(r – Pearson’s correlation coefficient; *p* <0.05; *Group A* women who achieved pregnancy; *Group B* women in whom pregnancy was not achieved; *E2/f* estradiol per ≥17 mm follicle, *FSH* follicle-stimulating hormone, *LH* luteinizing hormone, *AMH* anti-Müllerian hormone, *tF* time of the first frame in which both pronuclei could be observed, *tC* the frame with the last observation of both pronuclei, *t1-t9* times for corresponding numbers of cells, *tM* the first frame in which the embryos were compacting into the morula stage, *tB* the frame in which a crescent-shaped area began to emerge from the morula)

In group B, weak negative correlations were found between AMH levels and the following stages: t1 (*r* = −0.197, *p* = 0.018), t3 (*r* = −0.221, *p* = 0.008), t4 (*r* = −0.237, *p* = 0.004), t6 (*r* = −0.185, *p* = 0.026), and tB (*r* = −0.279, *p* = 0.001). Weak positive correlations were found for FSH level at tB stage (*r* = 0.281, *p* = 0.001) and for E2/f for t8 stage (*r* = 0.254, *p* = 0.002). For LH level, a weak negative correlation was noted only at tF stage (*r* = −0.190, *p* = 0.023). Weak negative correlations for estradiol levels were observed in the following time stages: tF (*r* = −0.214, *p* = 0.010), t3 (*r* = −0.166, *p* = 0.047), t4 (*r* = −0.225, *p* = 0.007), and tB (*r* = −0.234, *p* = 0.005).

### Prognostic value of AMH and FSH levels on the outcomes of IVF

**Fig. 6:**
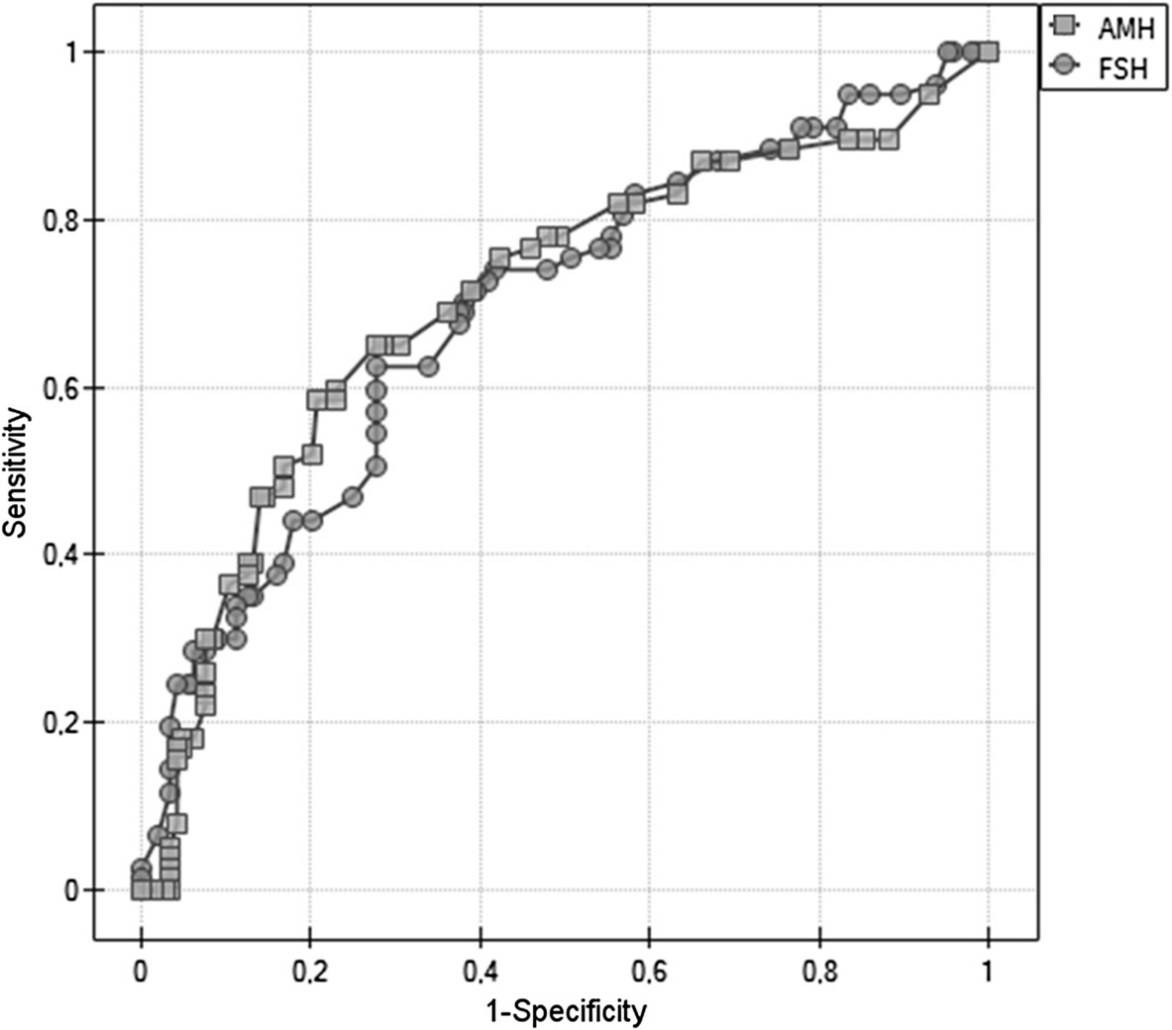
Receiver operating characteristic (ROC) curve showing the sensitivity and the 1-specificity of AMH and FSH for predicting the occurrence of pregnancy. (FSH – follicle-stimulating hormone; AMH – anti-Müllerian hormone)

The accuracy of using FSH and AMH levels to predict pregnancy after IVF was determined by ROC curve analysis (Fig. 6). An FSH level of 4.7 provides maximum discrimination between whether or not pregnancy will be achieved, with 28.57 % sensitivity and 93.75 % specificity. Similarly, an AMH level of 3.5 has 46.75 % sensitivity and 86.11 % specificity for indicating whether pregnancy will be achieved. The value of area under the curve (AUC) for FSH was 0.692, while the AUC value for AMH was 0.705. The assumed level for α was 0.05 and the obtained value for p was 0.597. There were no statistically significant differences between the curves for FSH or AMH (Z = 383, *p* = 0.702).

## Discussion

The dynamics of embryo development and IVF outcomes are conditioned by many factors. We evaluated the influence of gonadotropins on the timing of embryo development with use the time-lapse monitoring, and we compared hormones levels in women who achieved pregnancy and in women who did not achieve pregnancy with ICSI procedure. We are aware that other factors such as age, BMI, or quality of oocytes can affect embryo development and ICSI outcomes; however, in order to make the text more clear and understandable we decided to focus only on a few items.

Pregnancy, in our study, was achieved in almost 35 % of women (group A). This ratio is comparable with results obtained by other authors e.g. Kirkegaard et al. (31 %) and Polanski et al. (35 %) [16, 17]. Taking into consideration the kind of gonadotropin used, we noted that the pregnancy rate was slightly higher in women stimulated with hFSH than in women stimulated with rFSH; however, this difference was not statistically significant. The observations made by Flicori et al. were similar – they also noted higher frequency of pregnancies in women treated with hFSH than in women treated with rFSH, and their results also turn out to be statistically insignificant [6]. In turn, Kilani et al. obtained 35 % frequency of pregnancies both for hFSH-stimulated women and rFSH-stimulated women. However, they observed that hFSH treatment was associated with a more efficient response than stimulation with rFSH [7].

In our study, in women who achieved pregnancy almost all subsequent embryo development stages were observed earlier than in women who did not achieve pregnancy, but only part of these differences were statistically significant (tC, t1,t2, t4, and tB stages). When evaluating the impact of gonadotropins on embryos growth we noted faster development after stimulation with hFSH than after rFSH administration, but the differences were significant only at stages tC, t2, and t3 in women who achieved pregnancy and at stages tF-t4 and tB in women who did not achieve pregnancy. Slightly different results were obtained by Muñoz et al. – they noted that after administration of rFSH embryos showed better timing of development than after stimulation with hFSH or both rFSh and hFSH; however, these differences turned out to be not significant. Contrary to our study, they observed no significant differences at t2 stage between women stimulated with rFSH or hFSH or both rFSH and hFSH. Muñoz et al. used a different protocol for oocyte stimulation, but it seems that these divergences are rather due to using different types of gonadotropins - in the case of hFSH they used gonadotropins containing LH activity, and we used a medicament free of LH [18].

Analyzing hormone levels in the studied groups, we noted that FSH mean concentration was higher in the group of women in whom pregnancy was not achieved, contrary to levels of AMH, estradiol, and E2/f that were higher in women who achieved pregnancy. Other studies also proved that a high estradiol level improves embryos development and IVF outcome, expressed as number of retrieved oocytes, number of high-grade embryos, number of transferred embryos, and implementation rate [18–22]. Munoz et al. also assessed that better IVF outcomes are achieved when the concentration of estradiol exceeds 2000 pg/mL. In turn, Kara et al. indicated a 4000 pg/mL value [18, 22]. Our results seems to confirm the observation of Munoz et al. because the mean estradiol level in women who achieved pregnancy was about 2600 pg/mL, in contrast to women in whom pregnancy was not achieved with mean estradiol level about 1700 pg/mL. In the present study the higher estradiol level obtained in women who achieved pregnancy may be due to the hFSH stimulation that was applied to over 50 % of these women. As shown by Kilani et al., treatment with hFSH is associated with higher estradiol concentration than stimulation with rFSH [7]. Moreover, we observed negative correlations between estradiol level and timing of embryo development at stages t2, t4, and t5, which is in agreement with the results obtained by Muñoz et al. - they noted differences in the embryo dynamics at stage t5 and in cc2, dependent on estradiol level, which is the duration of the period as a 2-blastomere embryo (t3-t2) [18].

Endocrine markers thought to be useful in distinguishing good and poor responders for ovarian stimulation are, among others, FSH and AMH, but none of them is adequate for predicting pregnancy outcomes [23]. In our study AMH levels were significantly higher in women who achieved pregnancy than in women in whom pregnancy was not achieved, and the sensitivity and specificity of AMH in predicting pregnancy were 46.75 and 86.11 %, respectively. It was shown by other authors that a low level of AMH may cause cycle cancelation, lower mean implantation rate, or lower chances of ongoing pregnancy [24, 25]. On the other hand, even with undetectable AMH, pregnancy after transfer is possible. Therefore, as the author suggested, extremely low levels of AMH should not be the only cause of exclusion of a patient from attempting IVF. Then, usefulness of AMH for forecasting pregnancy indicated in the current study is slightly different from the observation of Reichman et al. [25]. We also tried to check which parameter – AMH or FSH – could be better in the prognosis of whether pregnancy will be achieved, but due to an assumed level for α at 0.05 and the obtained p value (0.597) we cannot prove a better predictive value of any one parameter. Hussain et al. also assessed the relationships between AMH and FSH in women undergoing IVF/ICSI, and they observed no difference in cycle cancellation, clinical pregnancy, and live birth/ongoing pregnancy between groups of women with different hormone levels [26].

Regarding LH level, we did not show significant differences in LH concentrations between the groups of women in whom pregnancy was or was not achieved. Our observation is supported by results of Ramachandran et al. They also showed no influence of LH level on IVF outcomes [27].

Recently it has been suggested that the genotype of receptor for FSH should be take into account for the pharmacological approach to infertility treatment with FSH because the response to FSH stimulation seems to be associated with genetic background [28]. However, more clinical data are necessary to warrant routine use of the FSHR isoforms as a diagnostic test.

## Conclusions

1. The kind of gonadotropin applied to stimulate ovaries impacts the dynamics of embryo development: in women stimulated with hFSH subsequent development stages were usually observed earlier than in women treated with rFSH.
2. There was no statistically significant difference in pregnancy rate between hFSH-stimulated women and rFSH-stimulated women.
3. The mean estradiol level was higher in women who achieved pregnancy than in women in whom pregnancy was not achieved, and its values negatively correlates with some time stages of embryo development.
4. AMH and FSH had greater impact on achieving pregnancy than other hormones, and the values of AMH and FSH in predicting pregnancy are similar.
